# Supplementary material for: Comparison of Clinical Manifestations, Treatments, and Outcomes between Vespidae Sting and Formicidae Sting Patients in the Emergency Department in Taiwan
Source: Int J Environ Res Public Health. 2020 Aug 25;17(17):6162. doi: 10.3390/ijerph17176162 (PMC7504193; doi:10.3390/ijerph17176162)
Supplement: Supplementary file 1 [file ijerph-17-06162-s001.zip › ijerph-894100-supplementary.docx]

Supplementary Materials

**Table S1.** Medication used to treatment in this study.

|  | **Drug Name** | **Dosage** |
| --- | --- | --- |
| IV or IM steroid | Hydrocortisone | 100 mg |
|  | Dexamethasone | 5 mg |
|  | Methylprednisolone | 40 mg |
| Oral steroid | Prednisolone | 5 mg |
| IV or IM antihistamine | Diphenhydramine | 30 mg |
| Oral antihistamine | Fexofenadine | 30 mg |
|  | Loratadine | 5 mg |
| Oral antibioitics |  |  |
|  | Cephradine | 500 mg |
|  | Cephalexin | 500 mg |
|  | Amoxillin | 500 mg |
|  | Amoxillin and clavulanic acid | 500 mg/125 mg |
| Oral analgesics | Acetaminophen | 500 mg |
|  | Ibuprofen | 400 mg |
|  | Diclofenac | 50 mg |
|  | Ketorolac | 10 mg |
| IV or IM analgesics | Ketorolac | 30 mg |
| H_2_ blockers | Cimetidine | 300 mg |

IV = intravenous; IM = Intramuscular.

|  |
| --- |
|  |
| \| **Temperature** \| **Correlation Coefficient** \| ***p*-value** \| \| --- \| --- \| --- \| \| Vespidae \| 0.81 \| <0.01 \| \| Formicidae \| 0.98 \| <0.01 \| |
| (**A**) |
|  |
|  |
|  |
| \| **Relative Humidity** \| **Correlation Coefficient** \| ***p*-value** \| \| --- \| --- \| --- \| \| Vespidae \| −0.403 \| 1.96 \| \| Formicidae \| −0.538 \| 0.07 \| |
| (**B**) |

**Figure S1.** (**A**) Scatter plot with number of cases vs. average temperature in two groups. (**B**) Scatter plot with number of cases vs. relative humidity in two groups.
